# The Treatment of Abscess in Pott's Disease

**Published:** 1895-11-09

**Authors:** A. H. Tubby

**Affiliations:** Assistant Surgeon and in charge of Orthopædic Department, Westminster Hospital; Surgeon to the National Orthopædic Hospital and the Evelina Hospital for Sick Children.


### Medical Progress and Hospital Clinics.

[*The Editor will be glad to receive offers of co-operation and contributions from members of the profession. All letters should be addressed to* The Editor, at the Office, 428, Strand, London, W.C.]

### THE TREATMENT OF ABSCESS IN POTT'S DISEASE.

By A. H. Tubby, M.S.Lond., F.R.C.S.Eng., Assistant Surgeon and in charge of Orthopædic Department, Westminster Hospital; Surgeon to the National Orthopædic Hospital and the Evelina Hospital for Sick Children.

(*Continued from page* 80.)

**Aspiration.**—It is evident that the evacuation by this method can only be of service when the contents of the abscess are entirely sero-purulent. But the contents of spinal abscess are very rarely so. In the great majority, large caseous clots are found. Aspiration fails to remove these. All it effects is to remove the more liquid and least harmful part of the pus. It is urged in favour of aspiration that it may favour absorption; by what means I know not. Repeated aspiration at one spot may "coax" the pus to that spot. The risk run in introducing septic material is by no means small, though theoretically nil.

**Aspiration with Injection of Fluids.**—It is difficult to know what it is hoped will be effected. If the conclusion with regard to aspiration is correct that its application is very limited and unsatisfactory, in so far that it leaves caseous masses behind, then I fail to see how the injection of carbolic acid, corrosive sublimate, sulphurous acid, peroxide of hydrogen, or iodoform solutions can be efficacious. Are we to expect that the caseous masses will be rendered inert, and the abscess walls cleansed, by a mixture of antiseptics, quite uncertain in their action? I think not. Apart from these speculations, one definite danger is attached to the injection of fluids into spinal abscesses, particularly iliac and lumbar. Bradford and Lovett[4] record a death of a boy of five from hip disease after washing out a small cold abscess with a few ounces of one to forty carbolic acid. I have witnessed dangerous collapse in a man, aged twenty-two, who had very extensive iliac abscess. It was aspirated, and a considerable amount of creolin solution injected. The patient, who had hitherto been taking the anæsthetic very well, suddenly became livid and pulseless. After some perseverance in methods of resuscitation, such as artificial respiration and the injection of brandy, the patient was brought round.

**Incision and Drainage with or without Washing Out the Cavity with Antiseptics.**—The character of the abscess differs very largely. It may be that we have to deal with a localised abscess which is pointing, in which the spinal disease is quiescent and the track from the spine healed; or, on the contrary, we may have to deal with one which tracks beneath fasciæ, which has numerous pockets connected with it, overflowing from time to time into the main cavity through minute openings, the sinuses being found with exceeding difficulty or not at all at the time of operation; or, finally, some may be almost entirely intra-abdominal and pelvic. The localised forms are readily dealt with, and do well; of the others, at the best the outlook is doubtful. One essential point in the treatment is strict antisepsis from first to last. As some abscesses go on discharging for months, and it may be for years, the maintenance of strict antisepsis until the last drop of discharge has ceased is problematical, and in some cases well-nigh possible. But such a result must be aimed at in all cases to avoid the deadly risk of septic infection of the abscess walls with the resulting profuse discharge, hectic fever, and lardaceous disease. It is interesting to note that Bradford[5] relates a case of tetanus following incision and drainage.

If the abscess be present on both sides, it is better to open them at once, since a small communication often exists and pus wells over from the unopened side.

Cervical abscesses are best opened from the side of the neck at the posterior border of the sterno-mastoid unless there is urgent dyspnœa and dysphagia calling for instant treatment from the pressure of a retro-pharyngeal abscess, when an exit for pus may be obtained through the posterior wall of the pharynx, the child being placed face downward, or with the head hanging well over the back of the table. Dorsal abscesses should be opened where they point, generally to one side of the middle line of the back. The incision for lumbar abscess is made along the outer side of the transverse processes and carried down through the quadratus lumborum till the sac is reached. In dealing with iliac and psoas abscesses, it is essential that the incision for drainage be away from the groin and upper part of the thigh. In children it is almost useless to expect to keep the discharge sweet when the incision is so near the genitals. Even when fluctuation is present below Poupart's ligament, an opening should be made in the lumbar region, if possible, and pus evacuated there. This plan has the advantages of securing efficient and safe drainage; and sequestra, if lodged in the upper part of the cavity, are easily removed. Such a piece of good fortune is rarely met with even by those who have considerable experience of spinal abscess. The only occasion when an opening near the groin is permissible is in conjunction with a lumbar opening to aid in clearing and thoroughly cleansing the cavity, the groin opening to be sewn up before the dressings are applied. In any case it is wise in dealing with children to cover the dressings wherever they may be with waterproof material to prevent contamination of them.

**The Method advocated by Treves, Barker, and Others.**—The object of this procedure is to place the treatment of spinal abscess on the same footing as that of caries of bone elsewhere, viz., removal of the abscess cavity. In 1882, Israel,[6] of Berlin, operating on an abscess in the lumbar region, removed part of the twelfth rib, scraped out the carious portion of the diseased vertebral body and opened up the vertebral canal, from which a quantity of pus escaped. In 1884 Treves read a paper before the Royal Medico-Chirurgical Society, urging that psoas abscesses should be evacuated through the loin, and he gave the steps of an operation by which the psoas muscle could be reached at the outer margin of the erector spinæ by means of a vertical incision cutting through the sheaths of that muscle and the quadratus lumborum. The muscle sheath is incised, and the vertebræ examined by continuing the operation on the deep aspect of the muscle. In one case portions of the diseased vertebræ were removed. Treves[7] quoted three operations, after all of which the patients recovered well.[8] The advances made by Mr. Barker[9] in the treatment of cases of tubercular disease of the hip, in which by means of scraping and flushing with hot water primary union was obtained, led to the application of this method to spinal abscesses. In his article[10] dealing with the question Barker says: "It is only lately that thorough evacuation has come to mean, not only the removal of its fluid contents, but also of all solid and semi-solid caseous or calcareous *débris*; and not only this, but of that lining of half-organised exudation material which covers the whole inner surface of the abscess, formerly called the 'pyogenic membrane.'" The method is as follows: "Taking the case of a large psoas abscess in which the bone lesion is apparently stationary, or perhaps healing, but where the pus is steadily increasing, an incision is made over the lower part through sound structures and the liquid pus evacuated. By means of the well-known flushing scoop (Barker's), hot water at a temperature of 103 deg. to 105 deg. Fahr. is sent into the cavity from a reservoir, and carries in its reflux the remaining contents of the abscess. The flushing scoop is then used as a scraper to dislodge the more solid portions of the caseous matter, which are washed out by the flow of hot water. The walls of the cavity are gently scraped until all the soft lining is removed and the water allowed to flow till it emerges clear from the cavity. The scoop is withdrawn, excess of water squeezed out from the sac, and sponges on sticks are used to dry out the last traces of moisture. Then two or three ounces of fresh iodoform emulsion are poured into the deepest parts of the abscess, sutures placed in position, all excess of emulsion squeezed out, the wound closed and dressed."[11] Several successful cases are quoted. G. A. Wright[12] has dealt with tubercular abscesses on the same lines, except that an ordinary Volkmann's spoon and solution of perchloride of mercury 1 in 3,000 were used. He gives notes of twelve cases of abscess, nine of tubercular joints, and three arising from spinal caries. The result was favourable in ten, including all the spinal cases. In a clinical lecture delivered by Treves,[13] at the London Hospital, on the "Treatment of Spinal and other Tubercular Abscesses," he gives details of eight cases. By means of the finger and sharp spoon and large quantities of perchloride of mercury solution (1 in 5,000) the cavity is entirely cleared, and the lining membrane is removed. "After the scraping and flushing have been persevered with until all the lining membrane appears to have been removed, then comes what I believe to be the most important part of the operation—the rubbing of the abscess wall with sponges, and the thorough drying of the cavity. . . . It is surprising what a quantity of inflammatory material in the form of the slimy lining membrane, and even cheesy pus, comes away upon the sponges."

The evacuation of pus from the spinal canal, and even from the posterior mediastinum and thoracic cavity has been effected during the performance of laminectomy, and has resulted in a more rapid cure than might otherwise have taken place.

**The Removal of Sequestra.**—The diseased bone may with comparative ease be removed if the laminæ, transverse processes, and ribs are affected. But it cannot happen often that large sequestra arising from the vertebral bodies are so accessible and so loose that a pair of forceps or the fingers working through a lumbar incision can dislodge them. Many attempts have been made, notably by Ashurst, to get rid of carious bone by cutting down on to diseased vertebræ and scraping with a Volkmann's spoon. This can only be done in the lumbar region, and then not without exceeding difficulty and danger; while the operation is not often of much benefit. Certain cases are on record attended by brilliant success, but we wish to have the failures submitted for opinion in the same way.

**Complete Removal of the Sac by Dissection.**—This is very rarely possible. Mr. Watson Cheyne alluded in his paper on the treatment of spinal abscess[14] to a case in which he was able to dissect out the sac, and remove the spinous processes, the wound healing by first intention. Mr. Symond, in the discussion, also spoke of a large abscess over the trochanter in which this had been done.

To sum up the treatment of spinal abscess: 1. Receding abscess and quiescent foci are best treated on the quiescent plan. 2. Aspiration is to be avoided, unless in the case of a small residual abscess following a previous attempt by the radical method to obtain primary union, and when the abscess is so deeply situated and in such immediate contact with serous membranes and viscera that further scraping is dangerous. 3. Cervical abscess should not be opened through the pharynx unless dyspnœa and dysphagia are urgent, but at the side of the neck. 4. Psoas and lumbar abscesses are best treated by the method of Barker, Treves, and others. 5. This method is more likely to be successful, if the bone disease be quiescent or healing. 6. Openings in or near the groin are not permissible in children, and drainage tubes are sources of trouble, both from the danger of septic infection and the risk of converting the track of the tube into a tubercular sinus. 7. The merits of the lumbar incision are great, but its application is limited, and very frequently an incision as for the ligature of the external iliac artery by Astley Cooper's method is preferable. 8. The possibility of removing large sequestra of bone by cutting down on the vertebral column is very problematical, and in a large number of cases no such proceeding is called for, the diseased bone being simply carious or caseous in its surface. 9. Dissection out of the sac is not often possible, but should be attempted when feasible.

[1] Brit. Med. Jour., 1892, Vol. II., p. 1,423. [2] Michel states that of 48 iliac and lumbar abscesses, pus was also found in the pelvis in 39. Nouveau Dict. de Med. and Chir. [3] Trans. Amer. Orthop. Assoc., Vol. IV., p. 169. [4] Bradford and Lovett, Orthopædic Surgery, p. 92., c.f.; also Fränkel Wien. Med. Wochensch, 1884, p. 34, and Vincent, Med. Press and Circ., 1887, Vol. XXIV., p. 529. [5] Trans. Amer. Orthop. Assocn., Vol. I., p. 11. [6] Berlin. Klin. Wochensch. 1882, No. 10. [7] Brit. Med. Jour., Jan. 12th, 1884. [8] Too much insistence, however, cannot be made as to the rarity of the cases in which it will be possible to remove portions of necrosed bone. [9] Brit. Med. Jour., Jan. 19th, 1889, and Nov. 1st, 1890; also Med. Chir. Trans., 1889 and 1891. [10] Barker, Brit. Med. Jour., 1891, Vol. I., p. 275. [11] Abridged from Mr. Barker's account. [12] Brit. Med. Jour., 1891, Vol. I., p. 905. [13] Ibid., 1892, Vol. I., p. 1,122. [14] Brit. Med. Jour., Vol. II., p. 1,422.

